# Compounds from the marine sponge *Cribrochalina vasculum* offer a way to target IGF-1R mediated signaling in tumor cells

**DOI:** 10.18632/oncotarget.10361

**Published:** 2016-07-01

**Authors:** Ana Zovko, Metka Novak, Petra Hååg, Dimitry Kovalerchick, Teresa Holmlund, Katarina Färnegårdh, Micha Ilan, Shmuel Carmeli, Rolf Lewensohn, Kristina Viktorsson

**Affiliations:** ^1^ Department of Oncology and Pathology, Karolinska Biomics Center, Karolinska Institutet, Stockholm, Sweden; ^2^ School of Chemistry, Raymond and Beverly Sackler Faculty of Exact Sciences, Tel Aviv University, Tel Aviv, Israel; ^3^ Science for Life Laboratory, Drug Discovery and Development Platform, Department of Organic Chemistry, Stockholm University, Stockholm, Sweden; ^4^ Department of Zoology, George S Wise Faculty of Life Sciences, Tel Aviv University, Tel Aviv, Israel

**Keywords:** sponge, small molecule, natural products, lung cancer, insulin growth factor receptor

## Abstract

In this work two acetylene alcohols, compound **1** and compound **2**, which were isolated and identified from the sponge *Cribrochalina vasculum*, and which showed anti-tumor effects were further studied with respect to targets and action mechanisms. Gene expression analyses suggested insulin like growth factor receptor (**IGF-1R**) signaling to be instrumental in controlling anti-tumor efficacy of these compounds in non-small cell lung cancer (**NSCLC**). Indeed compounds **1** and **2** inhibited phosphorylation of IGF-1Rβ as well as reduced its target signaling molecules **IRS-1** and **PDK1** allowing inhibition of pro-survival signaling. *In silico* docking indicated that compound **1** binds to the kinase domain of **IGF-1R** at the same binding site as the well known tyrosine kinase inhibitor **AG1024**. Indeed, cellular thermal shift assay (**CETSA**) confirmed that *C. vasculum* compound **1** binds to **IGF-1R** but not to the membrane localized tyrosine kinase receptor **EGFR**. Importantly, we demonstrate that compound **1** causes **IGF-1Rβ** but not Insulin Receptor degradation specifically in tumor cells with no effects seen in normal diploid fibroblasts. Thus, these compounds hold potential as novel therapeutic agents targeting **IGF-1R** signaling for anti-tumor treatment.

## INTRODUCTION

Marine organisms, in particular sponges (Porifera), constitute a rich source of pharmaceutical compounds and for the last 50 years it has remained the dominant phylum from which natural products with anti-tumor activity have been discovered [1, 2]. Pharmaceutical interest in sponges arose with the discovery of nucleoside analogue spongouridine from the marine sponge *Cryptotethia crypta* [3, 4]. This nucleoside analogue was used to construct cytarabine which today is one of the most commonly used anti-leukemia drugs [5, 6]. Another example is eribulin, a truncated synthetic version made from halichondrin B identified in the sponge *Halichondria okadai*. Indeed eribulin demonstrated efficacy in metastatic breast cancer [7, 8] and is clinically used. A recent example of a marine-derived drug is PM060184, a polyketide amide, that was isolated from the sponge *Lithoplocamia lithistoides* in 2006 and rapidly proceeded into phase I clinical trial [9, 10].

In non-small cell lung cancer (NSCLC) chemotherapy treatment efficacy is often hampered due to the ability of NSCLC cells to circumvent drug-induced cytotoxicity in various ways [11]. Progress in understanding molecular aberrant pathways of NSCLC has led to the development of agents that specifically target growth factor receptors or their downstream signaling components thereby blocking tumor cell proliferation capacity. The most advanced targets in this respect that are used clinically to combat NSCLC are the epidermal growth factor receptor (EGFR) tyrosine kinase and the fusion protein between EML4 (echinoderm microtubule-associated protein-like 4) and anaplastic lymphoma kinase (ALK) [12, 13].

The insulin growth factor-1 receptor (IGF- 1R), is another transmembrane receptor with tyrosine kinase activity found in NSCLC and other tumor types [14–18]. IGF-1R is found in cells as a tetramer with two extracellular localized α domains which are responsible for associating with ligand and two β subunits which apart from ligand binding also harbor the active kinase pocket [14–18]. The β subunits also harbor docking sites for different adaptor proteins which subsequently control downstream kinase signaling such as MAPK and Akt signaling [14–18]. IGF-1R can bind its natural ligands IGF-1 and IGF-2 either as a homodimer or as a heterodimer with Insulin receptor A/B (InsR A/B). In the latter complex, also insulin can act as ligand but with alteration in IGF-1-regulated signaling cascades as the major outcome (reviewed in [14, 15]).

Three main approaches for targeting IGF-1R/InsR have been explored: monoclonal antibodies towards either IGF-1R *per se* or heterodimeric IGF-1R/InsR, neutralizing antibodies towards the ligands IGF–1/IGF–2 and small molecules which targets the tyrosine kinase domain of IGF-1R and which act as antagonists of kinase activity either in a ATP-competitive or non-competitive way [14–16]. Therapeutic strategies towards IGF-1R might also influence InsR signaling and vice versa since there is a high similarity between IGF-1R and InsR when it comes to ligand binding, structure of kinase domain and downstream activated pathways and given that these receptors can form hybrid receptors [15, 19].

The IGF-1R/InsR signaling as an anti-tumor target has accordingly been studied in preclinical NSCLC models using either small molecule inhibitors towards the kinase domain or IGF-1R/InsR targeting antibodies [14-16, 19-24]. Thus we previously showed that blocking IGF-1R signaling in NSCLC cells *in vitro* by the Tyrosine kinase inhibitor (TKI) AG1024 inhibited downstream proliferative signaling via Akt and resulted in cell death [23, 24]. Similarly Kim et al., showed that a kinase inhibitor that targets both IGF-1R and InsR, OSI-906 (linsitinib), caused inhibition of cell proliferation notably in NSCLC with wt EGFR and wt K-Ras [22]. Monoclonal antibodies towards IGF-1R have similarly been studied in NSCLC and other tumor cell lines *in vitro* as well as *in vivo* in xenografts and revealed to have anti-tumor activity when used alone but more promptly when combined with IGF-1R TKI, radiotherapy or chemotherapy in which they are reported to cause clear IGF-1Rβ degradation [19-21, 25-28].

Therapeutic approaches targeting IGF-1R signaling have also been evaluated in NSCLC clinical settings but unfortunately with less success than observed in pre-clinical NSCLC models (reviewed in [14-18, 20]). Thus figitumumab (CP-751871), an IGF-1R targeting monoclonal antibody, was found to have about 30% overall response rate in NSCLC, but severe toxicity caused the trial to close prior to completion [29]. Another IGF-1R monoclonal antibody, dalotuzumab (MK-0646*)*, known to cause IGF-1R degradation in tumor xenografts and to have comparable efficacy as the small molecule OSI-906 (linsitinib) [30], was analyzed in a phase I/II trial where it was combined with the EGFR inhibitor erlotinib in unselected NSCLC patients [31]. No clear difference in efficacy compared to erlotinib alone was evident and the toxicity between both regimens was equal. Hence the view is that targeting IGF-1R signaling in the clinical setting of NSCLC in combination with either other targeted agents and/or chemotherapy will only be of benefit for a subset of NSCLC patients and ongoing research is aimed to reveal biomarkers that can enable NSCLC patient selection as pointed out in several recent reviews in the field [14-18, 20, 32].

By screening for anti-tumor compounds in marine sponges, we recently isolated and characterized two molecules from the Caribbean sponge *Cribrochalina vasculum* (family Niphatidae, order Haplosclerida) which possessed anti-tumor activity [33]. Both molecules are acetylene alcohols (3*R*)-icos-(4*E*)-en-1-yn-3-ol (compound **1**) and (3*R*)-14-methyldocos-(4*E*)-en-1-yn-3-ol (compound **2**) and were also previously reported to have anti-tumor activity [34–38].

Importantly, we demonstrated that both compounds **1** and **2** caused strong cytotoxic activity in tumor cell lines from NSCLC, small cell lung cancer (SCLC) and ovarian carcinoma (OC) but not in normal cell lines or primary cells tested e.g. human cardiomyocytes, human peripheral blood mononuclear cells, bronchial and retina epithelium and foreskin or lung fibroblasts [33]. We also found that both these acetylene alcohols triggered cell cycle arrest and activated intrinsic apoptotic signaling resulting in clear caspase 9- and caspase 3 activation with a simultaneous decrease in Akt and Erk proliferative signaling in NSCLC cells [33]. In this work we further studied the anti-tumor activity of these acetylene alcohols with the aim to identify potential targets in tumor cells. By gene expression analyses of compound **2** treated NSCLC cells inhibition of IGF-1R signaling network was evident suggesting IGF-1R as a possible target. Here we demonstrate, using different methods, that IGF-1Rβ is indeed a target of these *C. vasculum* acetylene alcohols in tumor cells. Thus, the *C. vasculum* compound **1** binds to IGF-1Rβ but not InsR or EGFR in NSCLC cells, and both compounds **1** and **2** impair IGF-1Rβ phosphorylation and cause IGF-1Rβ degradation thereby impairing oncogenic signaling in tumor cells resulting in prominent cell death.

## RESULTS

### Gene expression profiling of NSCLC cells reveals IGF-1R signaling as a candidate pathway of action upon treatment with *C. vasculum* derived compounds

To reveal anti-tumor action mechanisms of *C. vasculum* derived compounds and identify potential targets, Affymetrix gene expression profiling was used. For this purpose the NSCLC cell line U-1810 and normal fibroblasts WI-38 were exposed to compound **2** for 24 hours after which total RNA was extracted and subjected to Affymetrix-based gene expression and subsequent Ingenuity pathway analyses (IPA) (Supplementary Figure S1A). Comparison of altered genes in treated versus untreated NSCLC cells revealed more than 7,000 genes that displayed over 1.5-fold alteration in expression. Importantly, in normal fibroblasts WI-38 treatment with compound **2** did not result in significant alteration in gene expression as compared to untreated cells (Supplementary Figure S1B, S1C).

**Figure 1:**
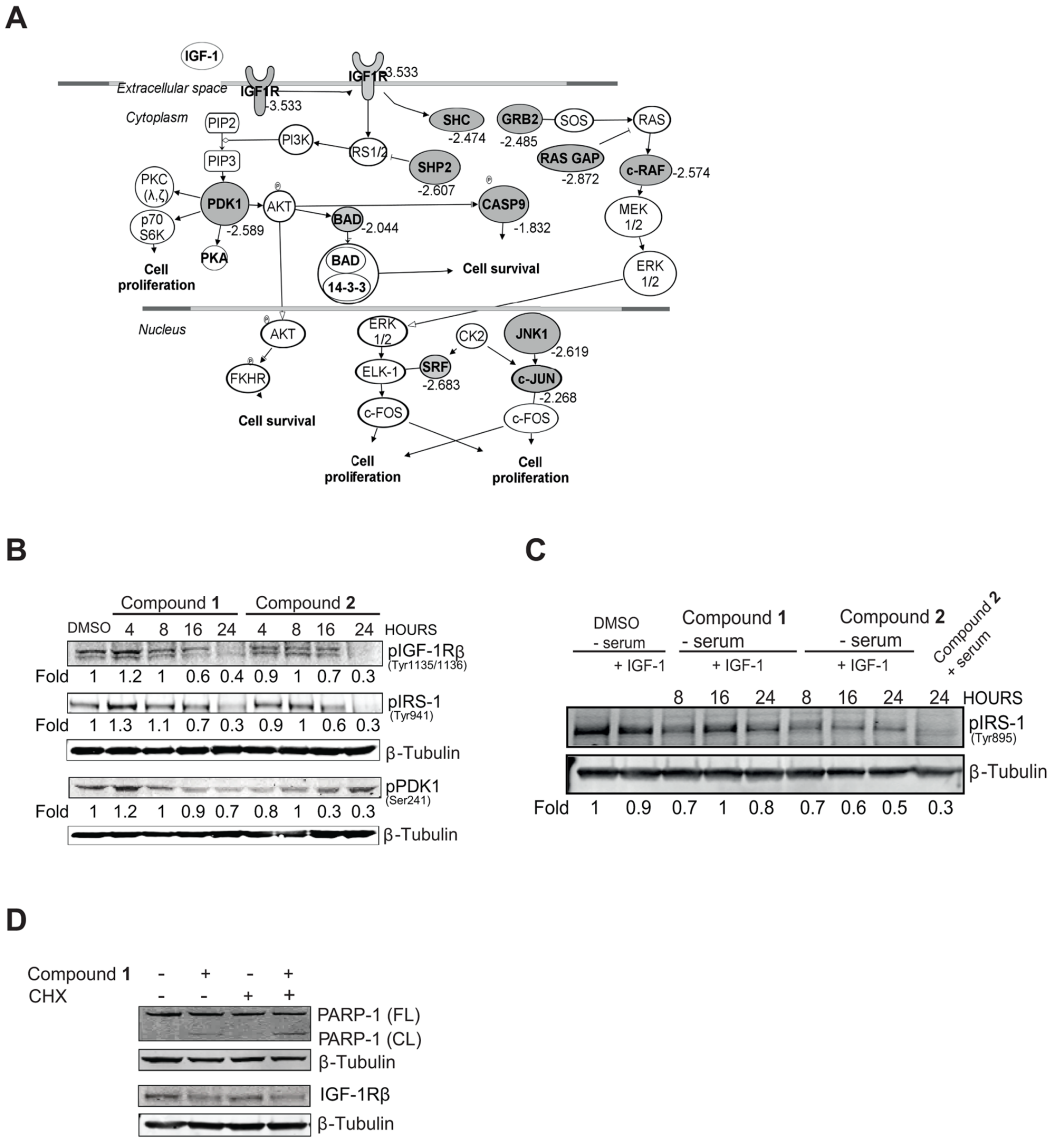
*C. vasculum* compounds block IGF-1R signaling **A.** NSCLC U-1810 or diploid fibroblasts WI-38 were treated with compound **2** (3 μmol/L) for 24 hours. Genes which showed differential expression (Fold to control=1.5, p<0.01) after treatment were analyzed with Ingenuity Pathway Analysis. An IGF-1R signaling network was found as one of the top scored networks. Significantly down-regulated genes are indicated in grey alongside their fold-down regulation expression values. **B.** NSCLC U-1810 were treated with compounds **1** or **2** for 4, 8, 16 and 24 hours at their IC_50_ (1.5 μmol/L and 15.1 μmol/L respectively or with equal volume of DMSO in the presence of serum-containing media for 24 hours and phosphorylation of IGF-1R, IRS-1 and PDK1 were analyzed with Western blot. Equal loading was confirmed by β-Tubulin. Expression was quantified by densitometry and is given as fold to DMSO-treated cells after correction for loading differences. **C.** NSCLC U-1810 cells were after 20 hours of serum starvation exposed to IGF-1 (50 ng/ml) alongside compounds as indicated in (B). Phosphorylation of IRS-1 was analyzed and β–Tubulin was used as loading control. To enable comparison, lane 9 contains U-1810 cells exposed as in (B). **D.** U-1810 cells were treated with CHX (0.5 μg/ml) for 8 hours followed by compound **1** (2 μmol/L) or DMSO for 16 hours. PARP cleavage and IGF-1R β deletion were examined. β-Tubulin was used to visualize equal loading.

To delineate altered signaling networks in response to compound **2** in the NSCLC tumor cells IPA was applied. The IPA analyses revealed an IGF-1R-controlled signaling network as one of the top ranked pathways (Figure 1A). Indeed, genes belonging to this signaling pathway including IGF-1R, PDK1, SHP2 and c-RAF showed a 2-to 4 fold decrease in expression in response to compound **2** (Figure 1A, grey symbols).

To further confirm that compound **2** was capable of altering an IGF-1R signaling network we made use of an 800 gene large signature identified in breast cancer (BC) cells upon IGF-1 treatment [39]. The genes of this signature (about 450 out of about 800) that were upregulated in response to IGF-1 were analyzed for overlap with gene alterations induced by compound **2**. Albeit the gene signature was obtained from BC cells and compound **2** was analyzed in NSCLC an about 60% of overlap was seen on gene identity level. Moreover, when these genes were loaded into IPA and the three top-ranked pathways were studied the overlap was almost 100% in terms of identity and most importantly, as expected, compound **2** caused a decreased expression of these genes further suggesting that compound **2** induced an IGF-1R signaling blockade (Supplementary Figure S1D). Thus gene expression analyses of compound **2** revealed IGF-1R signaling as a candidate network which was taken for further validation.

A number of IGF-1R signaling components were therefore next examined for their expression and/or phosphorylation levels in NSCLC U-1810 cells exposed to either of the two *C. vasculum* compounds **1** or **2** (Figure 1B). Indeed, NSCLC U-1810 cells exposed to compound **1** (1.5 μmol/L, corresponding to IC_50_) or **2** (15.1 μmol/L, corresponding to IC_50_) for 4, 8, 16 and 24 hours in serum-containing media showed impaired phosphorylation of IGF-1R β in a time dependent manner with a prominent, about 60% reduction in phosphorylation observed at 24 hours with either compound (Figure 1B). Similarly, both the insulin receptor substrate-1 (IRS-1) and PDK1, two components of the IGF-1R pathway, showed reduced phosphorylation in NSCLC U-1810 cells upon treatment with either of the two compounds (Figure 1B). We also examined the effect of compounds under serum-starvation condition with exogenous IGF-1 added with compounds and a 20-50% reduction in IRS-1 phosphorylation was evident (Figure 1C). For both compounds the effect on IRS-1 phosphorylation was less prominent than when cells were grown in serum (compare lane 8 with lane 9) (Figure 1C). To investigate how these *C. vasculum* derived compounds inhibited IGF-1R signaling, we examined if it was a result of inhibition of transcription as one could expect based on the alteration in gene expression of the IGF-1R signaling network. For that purpose the ability of compound **1** to induce apoptosis-associated cleavage of PARP-1 in the presence of cycloheximide (CHX), an inhibitor of *de novo* protein synthesis, was analyzed (Figure 1D). Pretreatment of NSCLC U-1810 cells with CHX did not reduce compound **1**-induced PARP-1 cleavage, suggesting that compound **1** is not likely to primarily mediate its IGF-1R blocking effect by alteration of transcription (Figure 1D).

### *C. vasculum* derived compounds inactivate IGF-1R-mediated signaling and cause IGF-1R receptor degradation in tumor cells

**Figure 2:**
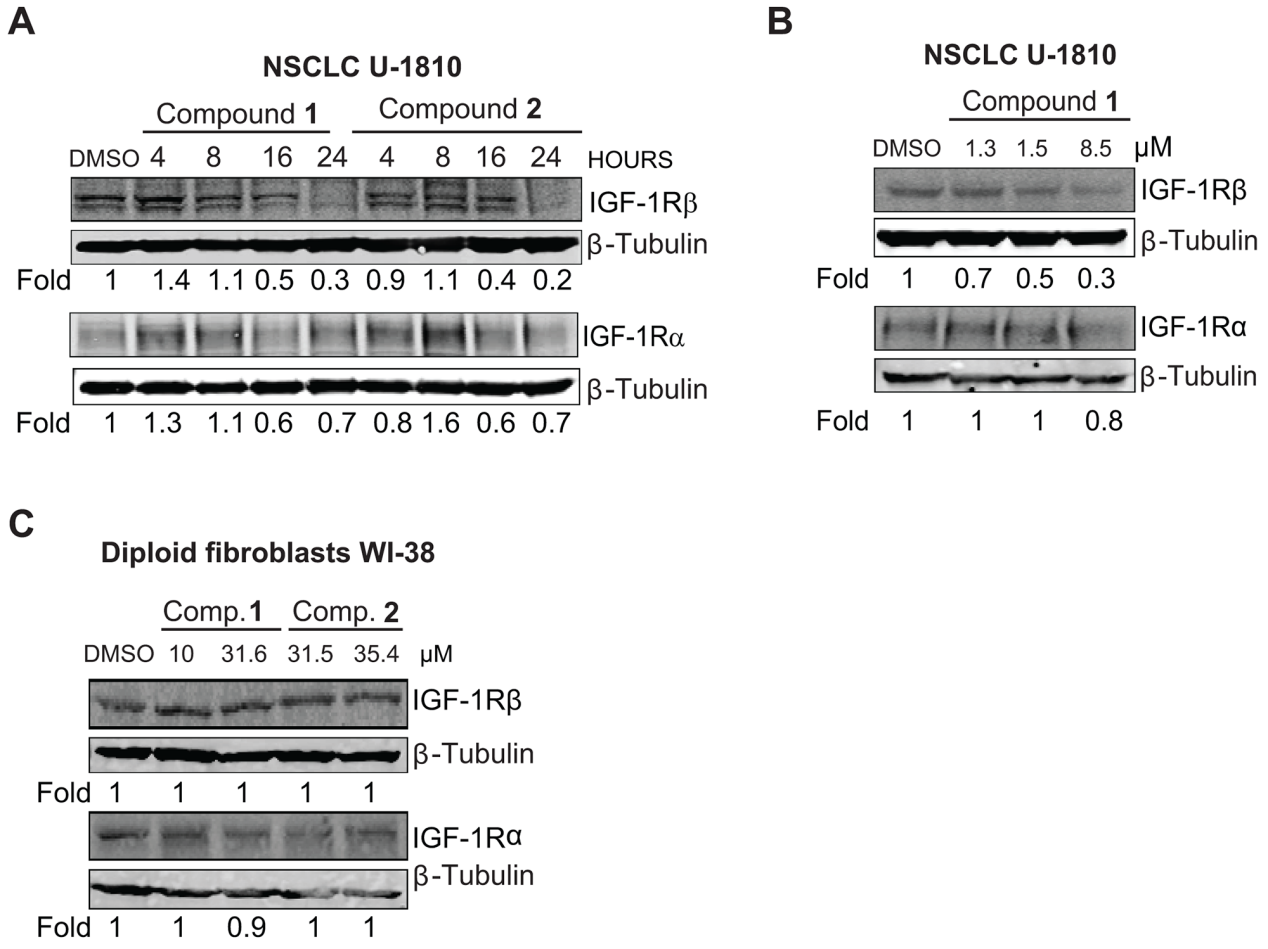
IGF-1R is depleted in NSCLC U-1810 but not in diploid fibroblasts WI-38 when treated with compounds 1 and 2 **A.** NSCLC U-1810 were exposed to IC_50_ of compound **1** (1.5 μmol/L) or **2** (15.1 μmol/L for 4, 8, 16 and 24 hours or with equal volume of DMSO for 24 hours and IGF-1R β and IGF-1R α total protein expression were examined. β-Tubulin was used to visualize equal loading. Expression was quantified by densitometry and is given as fold to DMSO-treated cells after correction for loading differences. **B.** NSCLC U-1810 were treated with IC_30_ (1.3 μmol/L), IC_50_ (1.5 μmol/L) and IC_70_ (8.5 μmol/L) of compound **1** for 24 hours or with equal volume of DMSO for 24 hours and total IGF-1R β and IGF-1R α were examined. **C.** Diploid fibroblasts WI-38 were treated with IC_50_ (10 μmol/L for compound **1** and 31.5 μmol/L for compound **2**) or IC_70_ (31.6 μmol/L for compound **1** and 35.4 μmol/L for compound **2**) for 24 hours and the change in total IGF-1R β and IGF-1R α protein level expressions were examined. β-Tubulin was used as loading control. Expression was assessed by densitometry and fold to DMSO-treated cells is given after correction for loading differences.

As a next step we analyzed if compounds **1** and **2** were instrumental in controlling IGF-1R protein expression levels using Western blot (Figure 2). Both compounds decreased IGF-1R β expression in NSCLC U-1810 cells in a time dependent manner and a 70-80% reduction in expression was seen 24 hours post compound addition when cells were grown in regular serum-containing media (Figure 2A). The effect on IGF-1R α expression was only minor and did not show time dependency (Figure 2A). By using different doses of compound **1** that reduced cell survival of NSCLC U-1810 cells by 30%, 50% and 70% respectively [33] and analyzing IGF-1R β levels we found that the magnitude in degradation paralleled cytotoxic activity (Figure 2B). In contrast the effect on IGF-1R α expression was only evident at the highest dose of compound **1**. The effect of compound **1** and **2** on IGF-1R β expression was also examined under serum-starvation conditions with exogenous IGF-1 added (Supplementary Figure S2). The reduction in IGF-1R β expression after treatment with either compound **1** or compound **2** was smaller than when cells grown in regular media yet at 24 hours a 30% and 50% reduction in IGF-1R β levels were found.

To ascertain if the observed effect of compounds **1** and **2** on IGF-1R β expression was specific for tumor cells the effect on IGF-1R β expression was also analyzed in normal diploid fibroblasts WI-38 (Figure 2C). Although IGF-1R β was expressed, exposure to the compounds did not result in degradation of either IGF-1R β or IGF-1R α in fibroblasts albeit they were treated with their IC_50_ or IC_70_ concentrations of the compounds for 24 hours (Figure 2C).

**Figure 3:**
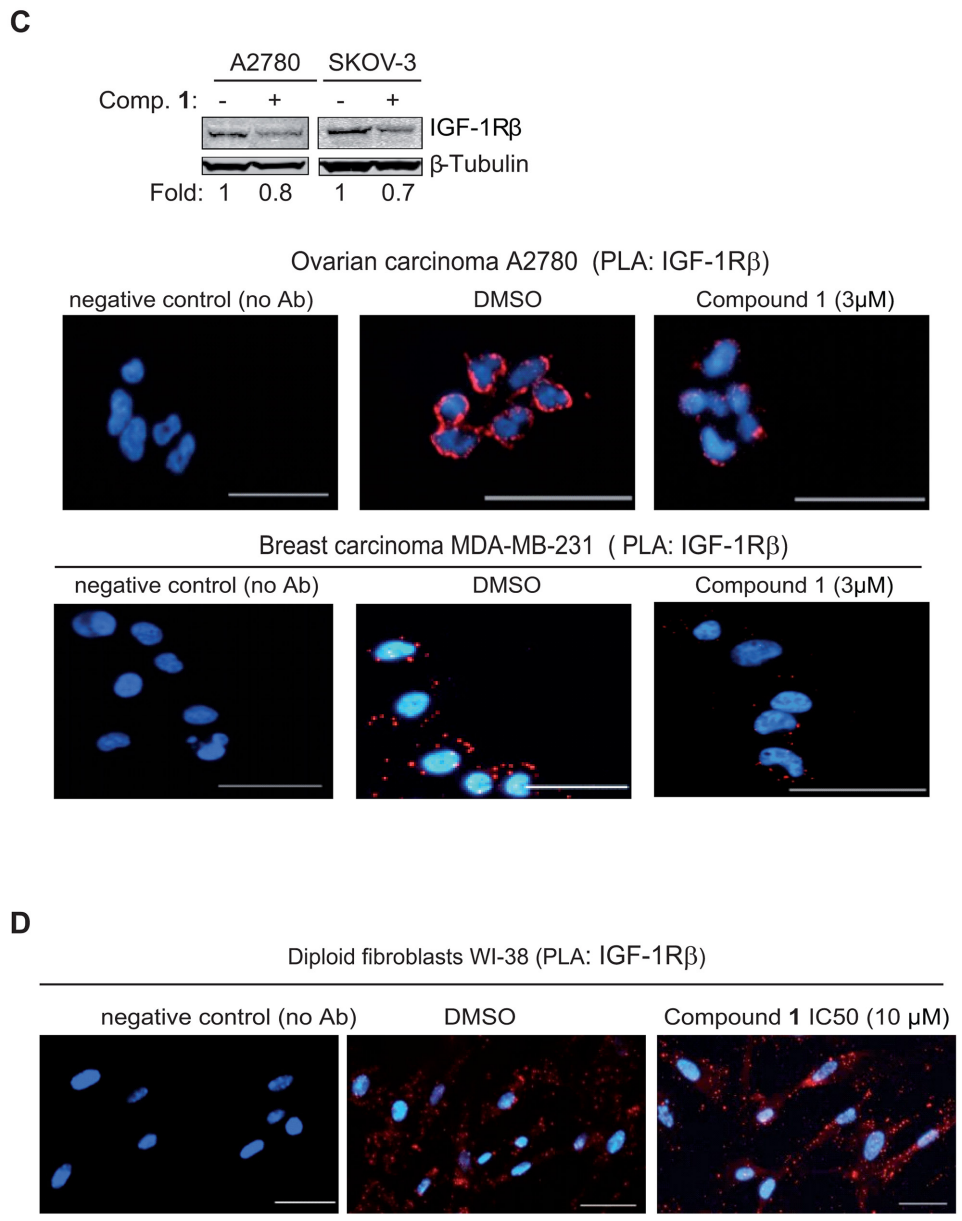
Proximity ligation assay demonstrates inhibition of phosphorylation and degradation of IGF-1R β in tumor cells but not normal diploid fibroblasts upon treatment with compound 1 **A.** NSCLC U-1810 cells were exposed to DMSO or IC_50_ of compound **1** (1.5 μmol/L) for 24 hours. Slides were stained with phospho-IGF-1R β (Tyr1135/1136) and total IGF-1R β antibodies in PLA (red) and nuclei counterstained with DAPI (blue). As a technical control for PLA, DMSO sample without the primary antibodies was used. Scale bars, 50 μm. **B.** *Left*: NSCLC U-1810 cells were exposed to DMSO or IC_30_ (1.3 μmol/L), IC_50_ (1.5 μmol/L) or IC_70_ (8.5 μmol/L) of compound **1**, AG1024 (10 μmol/L) or IGF-1R α/β siRNA for 24 hours. Slides were stained with two different IGF-1R β antibodies in PLA (red) and with DAPI (blue) to visualize cell nuclei. Scale bars, 50 μm. *Right*: IGF-1R β expression of cells treated with IGF-IR α/β siRNA or nonTargeting siRNA was verified by Western blot. β-Tubulin was used to visualize equal loading. **C.** OC A2780, OC SKOV-3 and BC MDA-MB-231 were treated with IC_50_ of compound **1** or with equal volume of DMSO for 24 hours and expression of IGF-1R β was examined. *Upper panel*: Western blotting of IGF-1R β in which β-Tubulin was used to visualize equal loading. Resulting bands were scanned by densitometry and fold to DMSO-treated cells is given after correction for loading differences. *Lower panel*: Cells were stained with two total IGF-1R β interacting antibodies as in (B). Scale bars, 50 μm. **D.** Diploid fibroblasts WI-38 were exposed to IC_50_ of compound **1** (10 μmol/L) for 24 hours labeled as in (B).

To further validate that compound **1** influenced IGF-1 phosphorylation and IGF-1R β expression in tumor cells proximity ligation assay (PLA) was applied (Figure 3). First antibodies towards phosphorylated and total IGF-1R β were used on NSCLC U-1810 cells treated with an IC_50_ concentration (1.5 μM) of compound **1** for 24 hours (Figure 3A). In DMSO treated cells PLA generated abundant signals demonstrating phosphorylation of IGF-1R β (Figure 3A, *middle panel)*. In contrast, in compound **1**-treated cells only faint signals remained suggesting a clear effect of compound **1** on IGF-1R phosphorylation (Figure 3A, *right panel*). When the effect of compound **1** was evaluated by PLA in WI-38 fibroblasts it was evident that the level of phosphorylated IGF-1R β in non-treated cells was much less than observed in NSCLC U-1810 cells and more importantly, the same low level of signals remained after compound **1** treatment (Supplementary Figure S3A).

PLA was also used to confirm degradation of IGF-1R β upon compound **1** treatment in tumor cells (Figure 3B–3C). In this setting antibodies against two different epitopes of IGF-1R β were applied thereby increasing the specificity and sensitivity in detection of IGF-1R β degradation. To show authenticity of the method IGF-1R α/β were depleted by siRNA in NSCLC U-1810 cells and the depletion was confirmed using Western blotting (Figure 3B, *lower right panel*). Indeed the PLA analyses revealed that IGF-1R β mediated signal was abolished in IGF-1R siRNA treated NSCLC U-1810 cells, showing the reliability of the method (Figure 3B, *1^st^*panel, 2*^nd^*row**). Importantly, whereas DMSO-treated NSCLC U-1810 cells expressed abundant IGF-1R β (Figure 3B, *1^st^*panel, 1*^st^*row**) treatment with compound **1** at different doses all resulted in decreased PLA signals indicating reduced IGF-1R β expression with a dose dependency seen in the degradation pattern (Figure 3B, *compare 2^nd^*and 4*^th^*panel, 1*^st^*row**). Interestingly, when cells were exposed to AG1024, a kinase inhibitor of IGF-1R at an IC_50_-dose IGF-1R β signals still remained illustrating that this TKI is not completely depleting IGF-1R β from the cells (2^nd^ panel, 2^nd^ row). PLA was also applied to study kinetics of IGF-1R β degradation (Supplementary Figure S3B). It was evident that the degradation starts already 4 hours post addition of compound **1** with a further increase over time. It is important to notice that 2 or 4 hours treatment with compound **1** had no influence on viability of cells demonstrating that degradation of IGF-1R was not a consequence of cell death (Supplementary Figure S4).

We also examined if compound **1** caused degradation of IGF-1R β in multiple tumor cell lines of different origin and in normal diploid fibroblasts WI-38 (Figure 3C–3D). We already showed that compound **1** induces significant cytotoxicity in ovarian cancer cell lines i.e. OC A2780 (IC_50_ after 72 hours of treatment 1.8 μmol/L) and SKOV-3 (IC_50_ after 72 hours of treatment 2.1 μmol/L) [33]. For breast cancer (BC) MDA-MB-231 cells IC_50_ after 72 hours of treatment was 1.8 μmol/L (IC_30_ 0.9 and IC_70_ 2.7 μmol/L) (data not shown). When ovarian cancer cells SKOV-3 and A2780 were exposed to 3 μmol/L of compound **1** for 24 hours a clear decrease in IGF-1R β expression was evident (Figure 3C, *top panel*). The PLA analysis also revealed that there was a decreased level of IGF-1R β in OC A2780 as well as in BC MDA-MB-231 (Figure 3C, *bottom panel*). PLA on IGF-1R β in diploid fibroblasts WI-38 also confirmed the Western blot data and demonstrated IGF-1R β expression albeit at a lower level than observed in NSCLC U-1810 cells (Figure 3D). Importantly, even though WI-38 fibroblasts were treated with IC_50_ concentration of compound **1** they still maintained IGF-1R β expression (Figure 3D). Taken together the results show that degradation of IGF-1R β is a specific action mechanism of the *C. vasculum* derived compound **1** in tumor but not in normal cells.

### *C. vasculum* derived compounds do not influence InsR β expression

**Figure 4:**
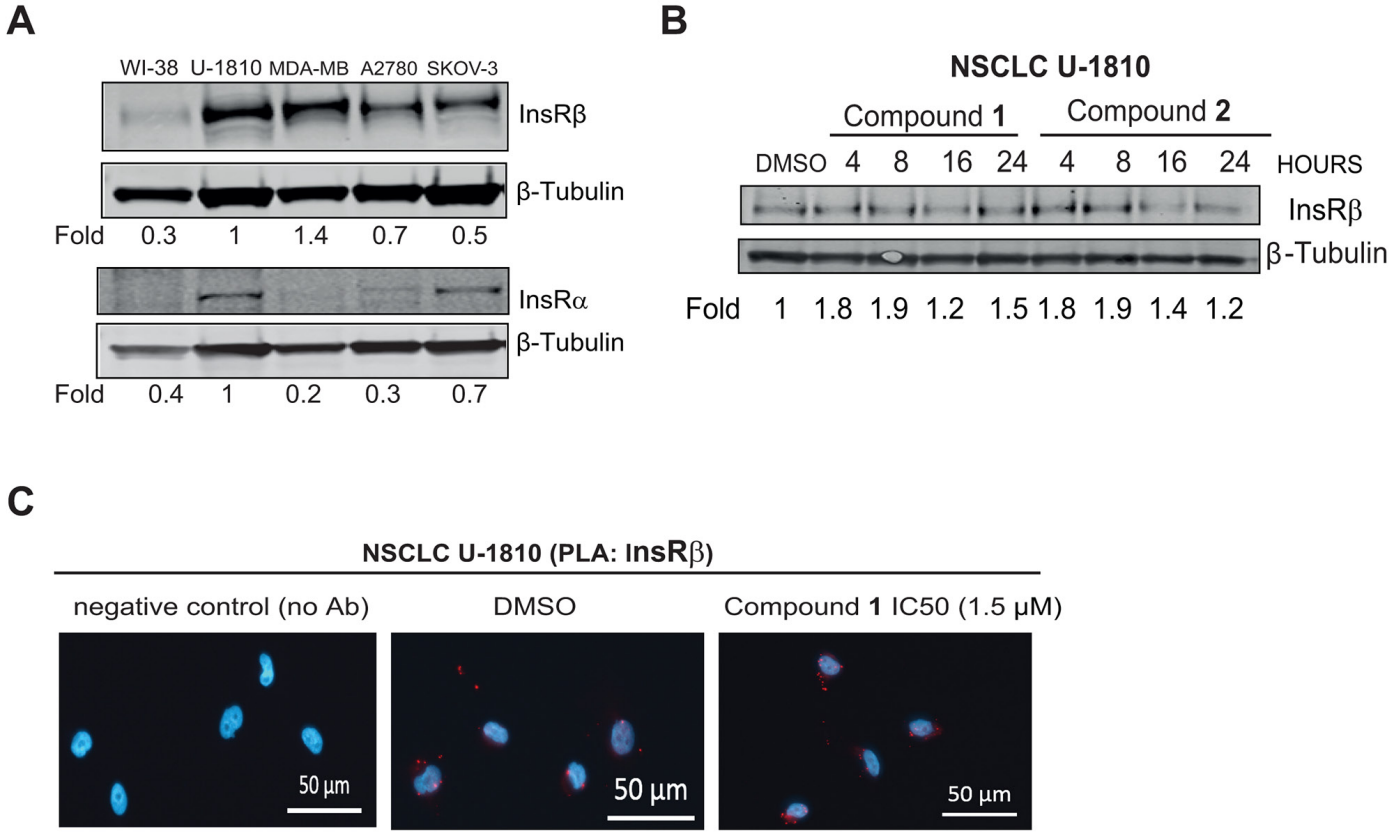
*C. vasculum* derived compounds cause minor effect on InsR expression in tumor cells **A.** Western blot analyses of InsR α and InsR β in untreated diploid fibroblasts WI-38, NSCLC U-1810, breast cancer MDA-MB-231 and ovarian carcinoma A2780 or SKOV-3. β-Tubulin was used to visualize equal loading. Resulting bands were scanned by densitometry and fold to DMSO-treated cells is given after correction for loading differences. **B.** NSCLC U-1810 were treated with IC_50_ of compound **1** (1.5 μmol/L) or **2** (15.1 μmol/L) for 4, 8, 16 and 24 hours or with equal volume of DMSO for 24 hours and InsR α and InsR β expression were examined. β-Tubulin was used to visualize equal loading. Resulting bands were scanned by densitometry and fold to DMSO-treated cells is given after correction for loading differences. **C.** NSCLC U-1810 cells were exposed to IC_50_ of compound **1** (1.5 μmol/L). Slides were stained with two different InsR β antibodies in PLA (red) and DAPI (blue) was applied to visualize cell nuclei. Scale bars, 50 μm.

The kinase domain of IGF-1R and InsR has similar structure and accordingly existing TKIs targeting IGF-1R have in most cases been shown also to influence InsR kinase signaling (reviewed in [14-18, 20]). One may therefore also consider that the observed effect of the compounds is a result of alteration in InsR expression levels. To address this, we first analyzed InsR α and InsR β expression in untreated NSCLC U-1810, breast- and ovarian cancer cell lines as well as in diploid fibroblasts WI-38 (Figure 4A). Results showed that InsR were abundantly expressed in NSCLC U-1810, breast and ovarian cancer cell lines but not in diploid fibroblasts. Next we analyzed how the compounds influence InsR β expression levels in NSCLC U-1810 (Figure 4B–4C). Western blot analyses revealed that compounds **1** and **2** did not reduce InsR β levels in NSCLC U-1810 cells as indicated by densitometric quantification (Figure 4B). Similarly in PLA, InsR β was found in DMSO-treated cells and the same magnitude of signal was found also when compound **1** was applied with a dose that caused a 50% block in cell survival (Figure 4C). The effect of compound **1** under serum-starvation condition after addition of exogenous insulin was also studied in NSCLC U-1810 cells (Supplementary Figure S5). No reduction in phospho-IRS-1 levels after compound **1** treatment was observed under these conditions. Thus results support that compound **1** does not target InsR β.

### CETSA analysis reveal that compound 1 bind to IGF-1Rβ but not EGFR in tumor cells

We reasoned that the observed effect of the compounds on IGF-1R β degradation could either be an indirect effect or be attributed to direct binding of compounds to IGF-1R β, which may alter its stability and cause degradation. We first assessed if compound **1** entered tumor cells by measuring compound **1** accumulation over time by LC- MS (Supplementary Figure S6). Results revealed that compound **1** indeed entered NSCLC U-1810 cells in a time- and concentration dependent manner.

**Figure 5:**
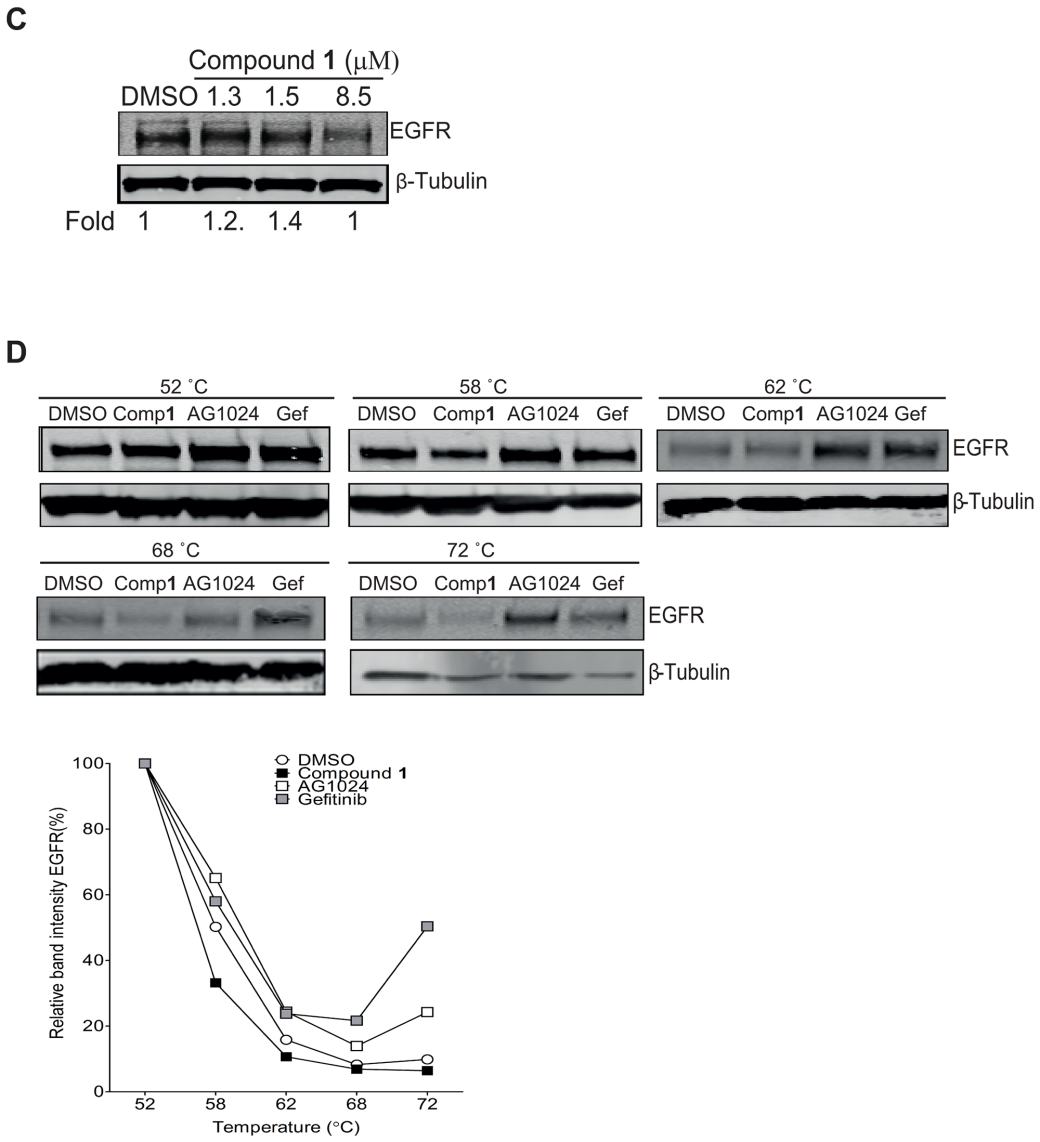
Confirmation of compound 1 binding to IGF-1R β but not EGFR by Cellular Thermal Shift (CETSA) technology **A.** NSCLC U-1810 cells were exposed to 50 μmol/L of compound **1** or AG1024 (positive control) or equal volume of DMSO for 2 hours, harvested and incubated in designated temperatures for 3 minutes. *Upper panel*: Western blot analysis of IGF-1R β in which β-Tubulin was used as marker for equal loading. *Lower panel*: Bands were quantified by densitometry and plotted against temperature after adjusting for loading differences. **B.** NSCLC U-1810 cells were exposed to compound **1**, harvested and incubated for 3 minutes at 58°C *Upper panel*: The presence of IGF-1R β was analyzed by Western blotting in which β-Tubulin served as marker of equal loading. *Lower panel*: Expression of IGF-1R β were examined by densitometry and after correction for loading differences plotted against concentration. **C.** NSCLC U-1810 were treated with IC_30_ (1.3 μmol/L), IC_50_ (1.5 μmol/L) and IC_70_ (8.5 μmol/L) of compound **1** or with equal volume of DMSO for 24 hours blotted for EGFR levels. β-Tubulin served as control for equal loading. Bands were quantified and are given relative to DMSO-treated cells. **D.** Cells were treated with 50 μmol/L of compound **1**, 50 μmol/L of gefitinib (Gef; positive control), AG1024 (negative control) or equal volume of DMSO for 2 hours, harvested and incubated with designated temperatures for 3 minutes. *Upper panel*: The presence of EGFR was analyzed by Western blotting in which β-Tubulin served as equal loading marker. *Lower panel*: EGFR expression were quantified by densitometry and plotted against temperature.

Next we set out to analyze if compound **1** directly binds to IGF-1R β by applying Cellular Thermal Shift Assay (CETSA), a method which relay on the concept that binding of a ligand or small molecule to its protein target results in target protein stabilization at a certain permissive temperature [40, 41]. For binding assessment using CETSA with respect to IGF-1Rβ NSCLC U-1810 cells exposed to compound **1** or the IGF-1R/InsR TKI AG1024, and the subsequent IGF-1R β stabilization were analyzed (Figure 5A–5B). While IGF-1R β bands completely disappeared in cells treated with DMSO at 52°C, they remained in cells treated with AG1024 and more importantly in cells treated with compound **1** for up to 62°C (Figure 5A). The significant shift in melting temperatures of IGF-1R β protein and hence stabilization upon addition of the compound **1** or AG1024 indicate indeed that both compounds bind to this protein. Importantly, treatment with 50 μmol/L concentration of either compound **1** or AG1024 for 2 hours did not cause any alteration in cell viability (data not shown), suggesting that the observed effects by compound **1** on IGF-1R β is not a consequence of general membrane destruction and/or permeability.

In the isothermal dose-response fingerprint (ITDRF_CETSA_) experiment, an increased stability of IGF-1R β protein in parallel to increasing concentrations of compound **1** was also evident (Figure 5B), further confirming that compound **1** binds to IGF-1R β and also illustrating the existence of a dose dependence in the interaction of compound **1** with IGF-1R β.

To confirm that compound **1** specifically binds to IGF-1R β and not to plasma membrane RTKs in general, the binding to EGFR, an RTK of relevance for NSCLC, was evaluated (Figure 5C–5D). First, the expression level of EGFR upon compound **1** treatment of NSCLC U-1810 cells was analyzed by Western blotting (Figure 5C). Importantly, in contrast to IGF1-R β little or no decrease in EGFR levels were evident even if a dose of compound **1** which blocked cell growth by 70% i.e. 8.5 μM was applied (Figure 5C). Next CETSA was used to evaluate the stabilization of EGFR by compound **1** in which the EGFR inhibitor gefitinib was used as positive control for stabilization (Figure 5D).

Gefitinib stabilized EGFR at temperatures even up to 72°C illustrating the functionality of the CETSA method for EGFR analyses (Figure 5D). Importantly, already at 62°C compound **1** failed to stabilize EGFR and the level of EGFR was similar as in DMSO-treated cells with a further degradation of EGFR seen at 68°C and 72°C (Figure 5D). Unexpectedly, AG1024 also stabilized EGFR under these conditions suggesting that it may either cross-react with EGFR or that stabilization of IGF-1Rβ/InsR also influences EGFR stability (Figure 5D). Thus, we conclude that compound **1** specifically binds to IGF-1R β under the conditions tested.

### *In situ* molecular docking results suggest binding of compound 1 to a similar site as AG1024 and cell experiments confirm a common interaction point within IGF-1R

**Figure 6:**
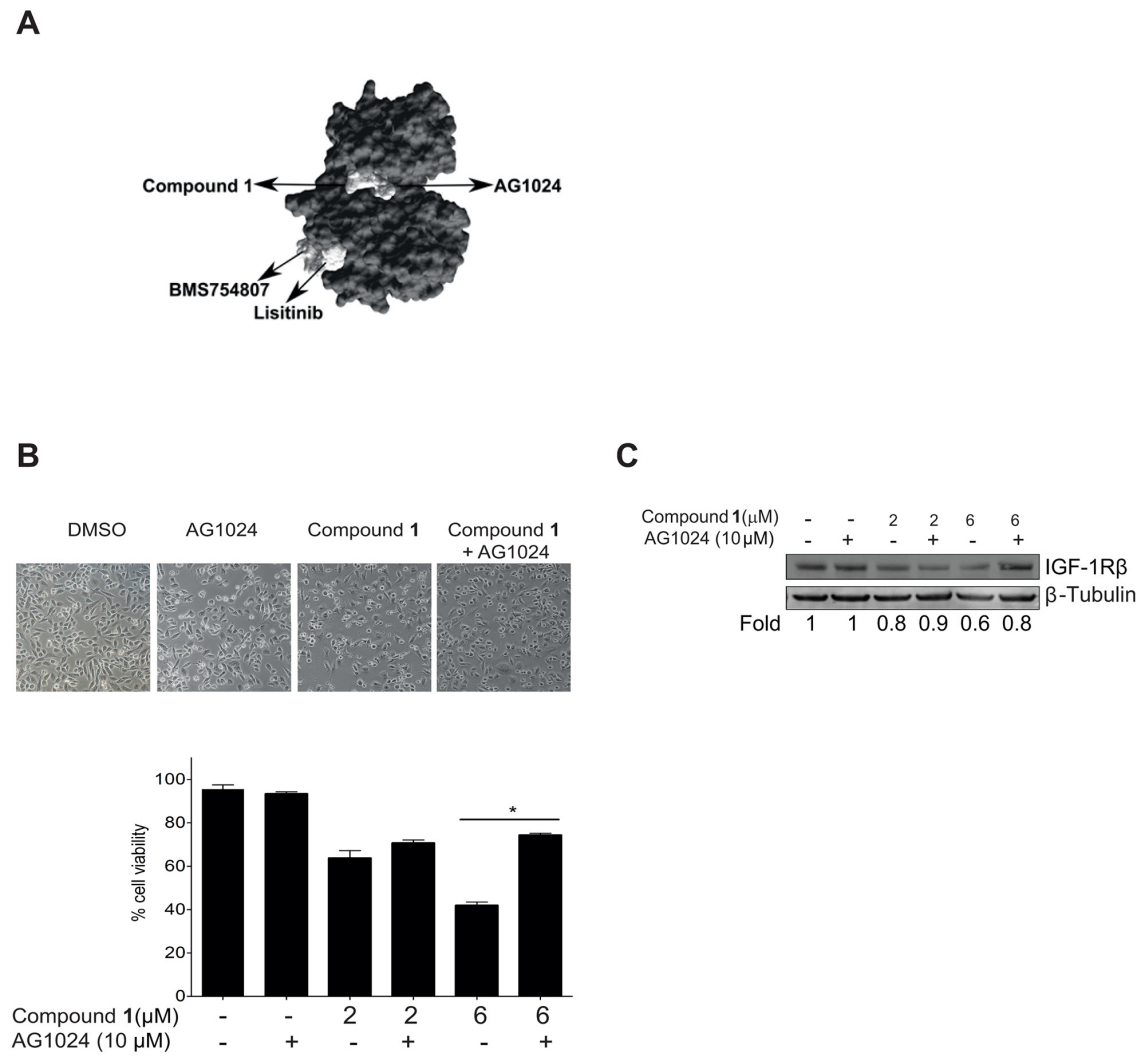
Compound 1 competes with AG1024 for binding to the IGF-1Rβ kinase domain **A.** The kinase domain structure of IGF-1R (PDB; 2ZM3), was used as a target molecule and compound **1**, AG1024, linsitinib (OSI-906) and BMS 754,807 were used as ligands in docking carried out with the SwissDock software program. The binding modes of the ligands that showed most favorable energy were visualized using UCSF Chimera molecular viewer. An arrow indicates the binding site of each compound. **B.** NSCLC U-1810 cells were treated with compound **1** (2 or 6 μmol/L) or AG1024 (10 μmol/L) or a combination for 16 hours and effect on cell viability was examined. *Top panel*: phase contrast images of cells, 10x magnification. *Bottom panel*: Quantification of viable cells in three independent experiments. Data presented are relative to DMSO-treated cells. *p<0.01. **C.** Effect on IGF-1Rβ depletion was examined with Western blotting in NSCLC U-1810 cells treated as in (B) in which β-Tubulin was used as marker of equal loading. Resulting bands were scanned by densitometry and fold to DMSO-treated cells is given after correction for loading differences.

In order to reveal putative interaction possibilities of *C. vasculum* compound **1** with IGF-1R, *in situ* docking of compound **1** to the IGF-1R kinase domain was examined [42, 43]. Docking data indicated that the possible binding site of compound **1** to the IGF-1R kinase domain was the same as for AG1024 which did not overlap with two other IGF-1R inhibitors linsitinib (OSI-906) and BMS 754,807 respectively (Figure 6A). In order to study if the observed common interaction point of compound **1** and AG1024 indeed was the case in tumor cells, NSCLC U-1810 cells were exposed to compound **1** alone or in combination with AG1024 for 16 hours, using a concentration of AG1024 which *per se* was not toxic (Figure 6B). Cells treated with a combination of AG1024 and compound **1** were less affected by compound **1** treatment than those treated solely with compound **1** (Figure 6B, left). Thus viability of U-1810 cells was significantly lower after treatment with 6 μmol/L compound **1** as compared to compound **1** in combination with AG1024 (Figure 6B, right). This suggests that once AG1024 is bound to IGF-1R compound **1** cannot compete for the binding site within IGF-1R. Importantly, assessment of IGF-1R β expression, revealed degradation when cells were treated solely with compound **1**, but when treatment was combined with AG1024, IGF-1R β was still expressed (Figure 6C).

## DISCUSSION

We previously reported that 3-hydroxyalkyl-4-ene-1-ynes isolated from the sponge *C. vasculum* are able to inhibit tumor (NSCLC, SCLC and ovarian) cell proliferation while having less effect on normal cells [33]. Moreover, we found that these compounds cause activation of mitochondria-mediated caspase-3 apoptotic signaling and block proliferative signaling via Ras/Raf/MAP kinase pathway and PI3K/Akt [33]. These results suggested that growth factor receptors could be targets of these *C. vasculum* compounds in tumor cells.

To search for relevant growth factor receptors, which could be responsible for the observed anti-tumor effect of these compounds, we performed gene expression analysis upon treatment with compound **2**. Interestingly, although analyses revealed more than 7,000 genes having significant alteration within tumor cells, none of the genes were affected in normal fibroblasts further illustrating a tumor selective action of these compounds. By employing Ingenuity pathway analyses on the altered genes, decreased IGF-1R signaling in response to treatment of tumor cells with compound **2** was evident. Moreover, we also compared our compound **2**-induced gene expression alterations in tumor cells with a published gene signature associated with IGF-1 response in breast cancer cells [39]. This comparison showed that about 60% of the upregulated genes in the signature of IGF-1 treated BC cells were in fact down regulated in our compound-regulated gene expression data of NSCLC cells. Hence further suggesting that *C. vasculum* compound **2** in fact targets the IGF-R pathway resulting in a block in this growth controlling cascade. Following these gene expression results, we accordingly found that IGF-1R as well as its downstream signaling molecules IRS-1 and PDK1 showed decreased phosphorylation upon exposure to *C. vasculum* compound **1** and **2** in NSCLC cells grown in serum-supplemented growth medium. Also under serum depletion with addition of exogenous IGF-1 IRS-1 showed decreased phosphorylation in cells treated with compounds **1** and **2** albeit of less magnitude than when cells were cultured in serum, which was the condition where we found these compounds to have specific anti-tumor activity.

One may speculate that serum conditions may favor IGF-1R phosphorylation *per se* allowing it to adopt a certain conformation which in turn permits the compounds to bind more thoroughly and inhibit phosphorylation of downstream targets. Alternatively, such serum conditions may also favor other receptor tyrosine kinases to have certain activity and to act on IGF-1R allowing it to adopt a conformation which allows compounds to bind. Given that we found normal fibroblasts to have less phosphorylated IGF-1R and to be non-responsive to compounds such a hypothesis is supported.

Importantly, we demonstrated that *C. vasculum* compound **1** caused a clear decrease in IGF-1R β protein expression in tumor cells of different origin i.e. NSCLC, breast- and ovarian carcinoma yet not in normal diploid fibroblasts, pointing towards a tumor selective action mechanism of compound **1** on IGF-1R β. By employing PLA we confirmed both a block in phosphorylation of IGF-1R in NSCLC cells but also degradation of IGF-1Rβ upon compound **1** treatment, the later also found in tumor cells of different origin. Hence our data support a mechanism in which the *C. vasculum* compound **1** binds IGF-1R resulting in degradation of the receptor, inhibition of downstream survival signaling cascades and prominent apoptosis induction.

It is known that the natural ligands of IGF-1R, IGF-1 and IGF-2 respectively also bind to InsRs [14–16]. Moreover, IGF-1R and InsR show high similarity in their kinase domains, share regulation of multiple kinase pathway downstream receptor activation and are also reported to form heterodimers with each other [14-16, 19, 20, 44]. Accordingly, it is recognized that multiple kinase inhibitors developed towards IGF-1R also target InsR (reviewed in [14, 15, 20]) while monoclonal antibodies are more specific towards IGF-1R but may as a consequence of IGF-1R internalization and degradation also alter hybrid IGF-1R:InsR expression in NSCLC and other tumor cells [19, 20, 25, 44].

Given these we therefore also evaluated compounds effect on InsR β expression in NSCLC cells with western blotting (compound **1** or **2**) and PLA (compound **1**). Neither with Western blot nor with PLA InsR β degradation was evident in NSCLC U-1810 cells upon treatment with compound **1**. Moreover, under serum starvation where insulin was added compound **1** did not cause reduction of phospho-IRS-1 further pointing out that compound **1** specifically targets IGF-1β over InsR β in this cell system. With respect to compound **2** we did not observe any InsR β degradation by Western blot whereas the effect on insulin-driven phospho-IRS-1 was less conclusive (data not shown). Yet in order to clearly show that InsR β is not a target of *C. vasculum* compounds an examination in tumor cells where InsR β is a major driver of growth is required, as reported to be the case in a subset of NSCLC cells [19].

IGF-1R β internalization and degradation have been shown to require phosphorylation of a tyrosine motif in the juxtamembrane region of IGF-1R β resulting in receptor internalization which is followed by ubiquitination and subsequent proteasomal/lysosomal degradation (reviewed in [45]). The later process is dependent on three E3 ubiquitin ligases Mdm2 (in combination with β-arrestins), c-Cbl and Nedd4, which are reported to have redundant and complementary roles in recycling, re-localization and degradation of the IGF-1R β. In our analysis of IGF-1R β degradation in cells grown under serum deprivation with exogenously added IGF-1 we observed decreased degradation upon compound treatment as compared to effects in tumor cells grown in serum conditions. One may speculate that this is attributed to loss of phosphorylation on certain sites within the IGF-1R β which is required for both compound binding and subsequent degradation. With respect to ligand-induced internalization of IGF-1 β it is reported to require phosphorylation of tyrosine 1250 [46] with the subsequent degradation requiring certain less defined IGF-1R β phosphorylation patterns to enable ubiquitination (reviewed in [45]). Moreover, IGF-1Rβ ubiquitination is also reported to be initiated from the intracellular part and to occur in a ligand-and kinase activity independent way via different adaptor proteins which associate with the intracellular domain of IGF-1R β (reviewed in [45]). Thus a complete elucidation on how compound **1** and compound **2** causes IGF-1R β degradation respectively would be interesting to further study using extensive mass spectrometry analyses of phosphorylation sites and interacting partners.

Results obtained with CETSA further support that compound **1** targets IGF-1R β as we found it to stabilize the receptor at temperatures that caused the receptor to be degraded in DMSO-treated cells. AG1024 a TKI known to interact with the active site of IGF-1R/InsR receptor [47, 48] served as a positive control in these experiments, indicating the functionality of the CETSA method. Thus CETSA analyses revealed that compound **1** directly binds to IGF-1Rβ in these NSCLC cells.

It is well known that tumor cells sometimes have endogenous production of growth receptor ligands causing an autocrine growth survival loop where the ligand activates the receptor. One may therefore speculate that NSCLC cells can endogenously produce IGF-2 causing an autocrine-signaling loop resulting in IGF-1R phosphorylation/activation. Albeit we found IGF-2 to have a higher expression in tumor vs. diploid fibroblasts we did not observe any major alteration in IGF-2 expression levels after treatment with compounds (data not shown) making this hypothesis unlikely. Moreover, as our CETSA results show that compound **1** binds directly to IGF-1R β it is unlikely that alteration of IGF-2 in tumor cells is the primary mechanism of action of the compounds.

One may consider that the compounds would also target other plasma membrane localized receptor tyrosine kinases and cause their degradation. Importantly, our CETSA analysis of EGFR upon compound **1** treatment of NSCLC cells did not indicate stabilization of EGFR illustrating that EGFR is not a target of compound **1** in these NSCLC cells and instead pointing towards specificity towards IGF-1Rβ for this *C. vasculum* compound.

*In vitro* docking experiments indicated that compound **1** could bind to the same site as AG1024 within the IGF-1R β kinase domain. These *in silico* data of binding of compound **1** and AG1024 were further strengthened, when tumor cells were treated with nontoxic concentration of AG1024 [48] in combination with compound **1**. AG1024 was found to be capable of reducing compound **1**-induced cytotoxicity pointing towards a common site of interaction within IGF-1R. Our interpretation of this finding is that when AG1024 is bound to the receptor it prevents the binding of compound **1**, abolishing the consecutive degradation of IGF-1R and thereby partially blocking cell death induced by compound **1**.

Targeting IGF-1R in NSCLC has in clinical settings been shown to be complicated and both small molecule inhibitors to IGF-1R/InsR as well as monoclonal antibodies towards IGF-1R have in unselected NSCLC patient cohorts failed to demonstrate efficacy and/or been associated with cytotoxicity [14-18, 20, 32]. Our *C. vasculum* compound **1** is a small molecule that potentially binds to the same kinase region as AG1024, yet compound **1** cause IGF-1Rβ degradation similarly as seen for IGF-1R Mab e.g. figitumumab (CP-751, 871) or MK-0646 [19, 20, 25-28, 30]. Thus we anticipate that our compound **1** will similarly to what was reported for IGF-1R TKI and IGF-1R Mab only be of benefit for a subset of NSCLC tumors. Yet its capacity to cause prominent cytotoxicity, block in Akt/MAPK signaling and degradation of IGF-1Rβ merits further analysis *in vivo* in NSCLC xenografts and in NSCLC patient cells where response biomarkers should be searched for upfront as recently been pointed out as a way to take IGF-R blocking therapies forward to clinical utility [14-18, 20, 32]. In particular, it will be interesting to examine if compound **1** could be used in combination with IGF-1R Mabs which also causes InsR degradation and/or TKIs which target EGFR signaling as these signaling events remain intact after compound **1** treatment. A comparison to already existing IGF-1R Mabs with respect to anti-tumor activity and toxicity should also be made.

In conclusion, in this study we show that both 3-hydroxyalkyl-4-ene-1-ynes isolated from sponge *C. vasculum* target IGF-1R, resulting in degradation of IGF-1R β in a tumor selective manner. For compound **1** we have also confirmed that degradation of IGF-1R is specific as this compound binds IGF-1R but not EGFR and does not causes InsR degradation. Adding these results to our previous study [33] makes us conclude that these compounds profoundly inhibit IGF-1R and impair MAPK and Akt pro-survival signaling, ultimately leading to activation of the intrinsic apoptotic pathway specifically in tumor cells which merits further *in vivo* validations in NSCLC xenografts.

## MATERIALS AND METHODS

### *C. vasculum* derived 3-hydroxyalkyl-4-en-1-ynes, chemicals and cell cultures

*C. vasculum* extracts were collected and purified to give (3*R*)-icos-4*E*-en-1-yn-3-ol (compound **1**) and (3*R*)-14-methyldocos-4*E*-en-1-yn-3-ol (compound **2**) as previously described [33]. Both compounds were dissolved in DMSO to give stock solutions with concentrations of 10 mg/ml.

The IGF-1R inhibitor Tyrphostin AG1024 (3-bromo-5-t-butyl-4-hydroxy-benzylidenemalonitrile) (Sigma Aldrich, Stockholm, Sweden) and the EGFR-inhibitor gefitinib (Selleckchem, Rungsted, Denmark) were dissolved in DMSO to stock solutions of 6.2 mmol/L and 10 mmol/L respectively. IGF-1 or Insulin (Sigma Aldrich) was reconstituted per manufacturers' instruction to give 50 ng/ml. Cycloheximide (CHX) (Sigma Aldrich) was dissolved in water to make 20 mg/ml stock solution. Stock solutions were kept at −20°C and diluted in cell culture media prior to use.

The human NSCLC U-1810 cell line [49] was a kind gift from Uppsala University. The ovarian cancer cell lines SKOV-3 and A2780 and the breast cancer cell line MDA-MB-231 were purchased from ATCC (Manassas, VA, USA). Lung fibroblasts WI-38 [50] was obtained from Coriell Cell Line Repository (Coriell Institute for Medical Research, Camden, NJ). Purchased cell lines were authenticated by cell banks of origin, using the short tandem repeat profiling and no authentication was done by the authors. U-1810 and A2780 cells were cultured in RPMI-1640 medium (Sigma Aldrich) supplemented with 2 mmol/L L-glutamine (Invitrogen, Stockholm, Sweden) and 10% heat-inactivated fetal bovine serum (FBS) (HyClone, Täby, Sweden). SKOV-3 cells were maintained in McCoy's 5A medium (Sigma-Aldrich) and MDA-MB-231 cells cultured in Leibovitz's L-15 supplemented as above. For lung fibroblasts WI-38 MEM medium (Invitrogen) supplemented with 15% FBS and 2 mmol/L of L-glutamine was applied.

### Gene expression profiling and analysis

For gene expression analysis, NSCLC U-1810 cells were treated with 3 μmol/L of compound **2** (in duplicates) or DMSO (in triplicates). Normal diploid WI-38 fibroblasts were treated in parallel with 3 μmol/L of compound **2** or DMSO (both in triplicates) to enable tumor-specific gene alterations caused by compound. For RNA isolation Trizol reagent (Invitrogen) was applied [51]. Samples were cleaned with Qiagen RNeasy Mini kit and labeled cDNA was made from total RNA using a standard Affymetrix protocol (Affymetrix, Santa Clara, CA, USA). For gene expression profiling the Affymetrix® whole transcript GeneChip® Human Gene 1.0 ST Arrays (Affymetrix) consisting of probes for 28,869 genes was applied. Initial processing of obtained gene expression data was made using the Affymetrix® GeneChip® Command Console® Software (AGCC) v 1.1 and Affymetrix Expression Console (EC) v 1.1 (Affymetrix) respectively. Further processing on the data was made using the probe logarithmic intensity error estimation (PLIER) and the perfect match GC composition-based background correction (PM GCBG) with global median used for normalization.

The similarity in gene expression between the different biological replicates was analyzed by principal component analysis (PCA) unit within Partek Genomics Suite (Partek Inc., St.Louis, Missouri, USA) in which the signal intensities from each probe were uploaded after background correction and normalization. The overall correlation of the different samples, according to the treatment and their gene expression, is presented. Hierarchical clustering of the genes was also made using the Partek software. Here, the expression levels in NSCLC U-1810 cells or WI-38 fibroblasts treated with DMSO were set to 1 and gene lists based on the up- and down-regulated genes over 1.5 fold (P< 0.05) by compound **2** was made. The list of treatment-induced differences in gene expression was further examined using Ingenuity Pathway Analysis (IPA, Ingenuity Systems, Inc., Redwood city, California, USA). IPA was applied to generate a top modified network associated with genes regulated upon treatment with compound **2**. To confirm effects of compound **2** on IGF-1 mediated signaling, similarity to a gene signature obtained from breast cancer cells treated with IGF-1 [39] was used. The number of overlapping genes from compound **2** treated NSCLC U-1810 cells and the upregulated genes in the IGF-1 signature from BC cells [39] were examined. The up-regulated genes in the signature were further uploaded into IPA and the overlap with top-regulated signaling network was explored.

### Cell viability assay

Compound **1**-induced cytotoxicity in MDA-MB-231 cells was examined by the 3-(4,5-dimethylthiazol-2-yl)-2, 5 diphenyltetrazolium bromide (MTT) assay as previously published [33]. Briefly, cells were seeded in 96 well plats and were after overnight incubation exposed to different concentrations of compound **1** or equal volumes of DMSO (v/v; negative control) diluted in fresh medium. Viability was examined at 72 hours post drug addition by adding MTT solution (Sigma-Aldrich, Germany) (0.5 mg/ml) for 4 hours at 37°C. Resulting formazan crystals were dissolved in stop solution (10% SDS and 0.01M HCl) and their absorbance quantified at 595 nm. Cell survival of compound treated cells was calculated based on absorbance relative to DMSO-treated cells set to 100%. The concentrations at which 70, 50 or 30% of cells were viable (IC_30_, IC_50_, IC_70_) were determined. The values presented are mean ± SEM from three biological replicates.

For short term exposure U-1810 cells were treated for 0.5, 1, 2 or 4 hours with 3 μmol/L of compound **1** and experiments were terminated immediately after end of exposure and cell viability examined by MTT assay as above.

For the other experiments studying the effects of the different compounds in fibroblasts WI-38, or cancer cells U-1810, SKOV-3 and A2780, concentrations which inhibited growth to 30, 50 or 70% (IC_30_, IC_50_, IC_70_) as previously described [33] was used.

### IGF-1R signaling analyses

For IGF-1R signaling analyses NSCLC U-1810 cells were treated for 4, 8, 16 or 24 hours with IC_50_ of either compound (compound **1**: 1.5 μmol/L, compound **2**: 15.1 μmol/L) or for 24 hours with compound **1** IC_30_ (1.3 μmol/L), IC_50_ (1.5 μM) or IC_70_ (8.5 μmol/L) or with equal volume of DMSO (negative control). Inhibition of IGF-1R or insulin-controlled phospho-IRS1 was also examined in U-1810 cells cultured in serum free media overnight and exposed to IGF-1 (50 ng/ml) or Insulin (10 nM) for 15 minutes. Ovarian carcinoma A2780 and SKOV-3 or breast cancer MDA-MB-231 cells were exposed to compound **1** (3 μmol/L) for 24 hours. Normal lung fibroblasts WI-38 were treated with IC_50_ (compound **1**: 10 μmol/L; compound **2**: 31.5 μmol/L) or IC_70_ (compound **1**: 31.63 μmol/L; compound **2**: 35.4 μmol/L) for 24 hours. For experiment in which binding of compound **1** to target was evaluated, NSCLC U-1810 cells were exposed to 2 or 6 μmol/L of compound **1** with or without AG1024 (10 μmol/L) for 16 hours. In cycloheximide experiment, U-1810 cells were pretreated with CHX (0.5 μg/mL) for 8 hours followed by treatment with 2 μmol/L of **1** for 16 hours.

### Western blot analysis

For Western blot analyses whole cell extracts were prepared as indicated [33] by using RIPA buffer (50 mM Tris-HCl, pH 7.4, 150 mM NaCl, 0.5 % Igepal, 5 mM EDTA and 0.1% SDS) complemented with protease and phosphatase inhibitor cocktail tablets (Roche Diagnostics AB, Stockholm, Sweden). 30-50 μg of total protein were mixed with reducing loading buffer (NuPAGE, Invitrogen) and resolved on Bis Tris 4-12% or Tris Acetate 3-8 % gels (NuPAGE, Invitrogen, Stockholm, Sweden) respectively. Electrophoresis was performed at 200V for 60 minutes in MES or MOPS running buffer (NuPAGE, Invitrogen) and proteins were transferred onto to polyvinylidene fluoride (PVDF) membranes (Hybond-C Extra, Amersham Biosciences) at 30 V for 90 minutes in transfer buffer (NuPAGE, Invitrogen) containing 10% methanol. For blocking the membranes were incubated in Odyssey blocking buffer (Li-Cor Biosciences, Germany) for 1 hour prior to overnight incubation with primary antibodies. Primary antibody binding was visualized with appropriate secondary antibody that was added for 1 hour at room temperature.

The following primary antibodies were used: phospho-IGF-1R β (Tyr1135/1136), IGF-1R β, phospho-PDK1 (Ser241), phospho-IRS-1 (Tyr895), EGFR (all from Cell Signaling Technology, MA, USA); IGF-1R α, phospho-IRS-1(Tyr941), PARP-1 (H250) (all from Santa Cruz Biotechnology, CA, USA), InsR α and InsR β (both from Abcam, Cambridge, MA, USA). β Tubulin (Sigma-Aldrich) was used as loading control. To reveal bands on the Odyssey platform IR-Dye-linked secondary antibodies (LI-COR Biosciences) were applied.

### Monitoring of compound binding to IGF-1R in cells using the cellular thermal shift assay

Binding of compound **1** to IGF-1R β was analyzed by cellular thermal shift assay (CETSA), a method which allows characterization of small target molecules and target interactions in cells and which relay on the principle that a target molecules will thermally stabilize the protein it interacts with [40, 41]. In this study stabilization of IGF-1R β or EGFR by compound **1** or IGF-1R or EGFR inhibitors AG1024 and gefitinib respectively was examined.

The melting curve for IGF-1R β was determined using U-1810 cells (2×10^7^) treated with 50 μmol/L of either compound **1** or AG1024 (positive control) for 2 hours. To ensure that treatment was not *per se* causing cell membrane disruption cell viability was quantified using trypan blue staining. Cell extracts for the CETSA analyses was obtained by trypsination followed by resuspension in PBS supplemented with protease inhibitor. The cell suspensions were incubated with designated temperatures (40-68°C) for 3 minutes in thermal cycler followed by 3 minutes at room temperature. After this, the samples were snap frozen and cells lysed with freeze-thaw method and vortexed briefly after each thawing step. In order to preserve all interactions of compounds with proteins the obtained cell lysates were incubated on ice and centrifuged (20,000 g for 20 minutes) to clear supernatants from undissolved cell debris.

To obtain data with respect to EGFR protein binding, experiments were carried out as above but using 50 μmol/L of either gefitinib (positive control), compound **1** or AG1024 (negative control) for 2 hours. Cell suspensions were incubated in temperature range 52-72°C.

Detection and quantification of IGF-1Rβ or EGFR was performed using Western blot as described above. Intensity of bands was plotted against temperatures used in experiment.

The procedure for determination of isothermal dose-response fingerprint (ITDRF_CETSA_) was carried out as described above but using different concentrations (10-100 μmol/L) of compound **1** and analyzing obtained cell pellets after incubation at 58°C for 3 minutes. Intensity of IGF-1Rβ bands was plotted against concentration of compounds used in the experiment.

### siRNA ablation of IGF-1R expression

To deplete IGF-1R expression in NSCLC U-1810 cells 100 nM IGF-IR α/β siRNA (Santa Cruz Biotechnology) was applied for 24 hours using Dharmafect. The Stealth RNAi Negative Control Duplexes (Invitrogen, Life technologies) was transfected in parallel to reveal off target effects. Ablation of IGF-IR α/β expression by siRNA was examined by Western blotting.

### Analysis of compound-induced IGF-1R depletion by proximity ligation assay (PLA)

To validate that phospho-IGF-1R, total IGF-1R β or total InsR β was depleted from cells treated with compound **1**, proximity ligation assay (PLA) method with Duolink II assay kit (Olink Bioscience, Uppsala, Sweden) was applied. NSCLC U-1810 cells were treated with DMSO, AG1024 (10 μmol/L) or compound **1** (IC_30_, IC_50_ or IC_70_) for 24 hours or compound **1** IC_50_ for 2, 4, 8 and 16 hours in the presence of media supplemented with serum. Cells in which IGF-1R expression was ablated by siRNA (see above) were used as a positive control. OC cells A2780 and BC cells MDA-MB-231 were exposed to 3 μmol/L of compound **1** for 24 hours or with equal amount of DMSO as negative control whereas fibroblasts WI-38 were treated with compound **1** for 24 hours with their IC_50_ concentration (10 μmol/L). After treatment cells were fixed on slides by incubating in 4% PFA solution for 20 minutes and subsequently dehydrated with increasing concentration of ethanol (70%, 80% and 100%). Slides were thereafter blocked in BSA (3%)/Triton-x 100 (0.1%)-buffer for 30 minutes. Two primary antibodies against IGF-1R from different species (Rabbit: IGF-1R β, Santa Cruz Biotechnology; SC-713 and Mouse: IGF-1R ab-1, Calbiochem; GR11, both diluted 1:100 in blocking solution) were added to the slides during a 1h incubation at room temperature to enable binding. Subsequently the PLA probes (diluted 1:5 in Antibody Diluent from kit) were applied onto the slides for another hour at 37°C followed by ligation and amplification according to manufactures instruction To visualize cell nuclei, slides were counterstained with DAPI in the mounting medium (Vector Laboratories) and resulting signals examined under the microscope. Fluorescent images were obtained using Axio Imager.Z2 (Zeiss) (containing a 100-W mercury lamp and a CCD camera (C4742-95, Hamamatsu)), which incorporates epi-fluorescence and transmitted-light bright field microscopy. Epi-fluorescence microscopy was used to detect and image fluorescent signals from hybridization of PLA probes using Texas Red filter and DAPI, respectively. Pictures were taken at 20x magnification. As a control, samples without the primary antibodies were used. Red fluorescent dots indicate the IGF-1R β – IGF-1R β interactions.

The same method was used to show InsR-β levels in NSCLC U-1810 cells treated with DMSO or IC_50_ of compound **1** for 24 hours. Two primary antibodies against InsR β from different species (Rabbit: InsR-β, Cell signaling; 3025 and Mouse: InsR β, Abcam; AB69508) were applied.

The degree of IGF-1R phosphorylation was also verified by PLA in U-1810 and WI-38 cells treated with IC_50_ concentration of compound **1** (1.5μmol/L for U-1810 and 10 μmol/L for WI-38) for 24 hours. Mouse antibody against IGF-1R (Calbiochem; GR11) and rabbit against phosphorylated IGF-1R (Cell signaling; 3024) were used for these analyses.

### Intracellular quantification of compound 1 by LC- MS (APCI)

To verify intracellular accumulation of compound **1** in tumor cells after treatment LC-MS (APCI) was used. For that purpose NSCLC U-1810 cells (4×10^6^/ml) were dispensed in 8 ml of cell culture media and treated with 3 μmol/L or 17 μmol/L of compound **1** or equal volume of DMSO. 0.5 mL of homogenous cell suspension was taken from the suspension immediately (t= 0 minutes) and at 10, 30 or 60 minutes after addition of compound **1**. Cells were centrifuged down (4 minutes, 1,400 RPM) in pre-cold centrifuge. To lyse and precipitate proteins from the cell pellet, 300 μl formic acid/water (0.1%v/v) and 1.3 ml of ice cold MeCN were applied to the sample which subsequently was incubated at −80°C for 16 hours followed by pre-cooled centrifugation (10 minutes, 10,000 RPM) to obtain supernatants without proteins. LC-MS (APCI) measurements on the supernatants were carried out by MetaSafe (Södertälje, Sweden) in which liquid chromatography analysis was run on a system consisting of an Accela LC (Thermo Fisher Scientific, San Jose, CA, USA) pump and a PAL auto-sampler. The following conditions were applied: Hypersil Gold column (100 × 2.1 mm ID, 1.9 μm particle size), eluting solution A: 0.1% formic acid/water, eluting solution B: 0.1% formic acid/MeOH, flow rate 0.4 mL/min. 30 μL of 17 μM sample and 50 μL of 3 μM were injected. The amount of compound was analyzed by an LTQ Orbitrap (Thermo Fisher Scientific) with APCI interface in positive ion mode with nitrogen used as sheath gas. Discharge current was 5 μA, FTMS full scan was in the range m/z 200-500 and resolution 7500. The Xcalibur 2.0 SR2 software (Thermo Fisher Scientific) was used for both acquisition and control of the MS and for processing obtained raw data. Peak area for each concentration and time point was measured and the concentration of compound **1** in sample treated with 17 μmol/L for 30 minutes was determined by spiking. Quantification of compound **1** in all other samples was related to the area of this sample and is given in μmol/L.

### Docking of compound 1 to IGF-1R kinase domain

To visualize the potential binding site of compound **1** to IGF-1R the SwissDock software program, which is based on the docking software EADock DSS [42], was applied. The kinase domain structure of IGF-1R (PDB; 2ZM3), was used as the target onto which compound **1** or the previously reported IGF-1R inhibitors AG1024, linsitinib (OSI-906) and BMS 754,807 were used as ligands. Binding modes with the most favorable energy were evaluated and visualized with UCSF Chimera molecular viewer [43].

## SUPPLEMENTARY MATERIALS FIGURES


