# Supplementary material for: Compounds from the marine sponge Cribrochalina vasculum offer a way to target IGF-1R mediated signaling in tumor cells
Source: Oncotarget. 2016 Jul 1;7(31):50258–76. doi: 10.18632/oncotarget.10361 (PMC5226581; doi:10.18632/oncotarget.10361)
Supplement: Supplementary file 1 [file oncotarget-07-50258-s001.pdf]

Compounds from the marine sponge *Cribrochalina vasculum* offer a way to target IGF-1R mediated signaling in tumor cells

SUPPLEMENTARY FIGURES

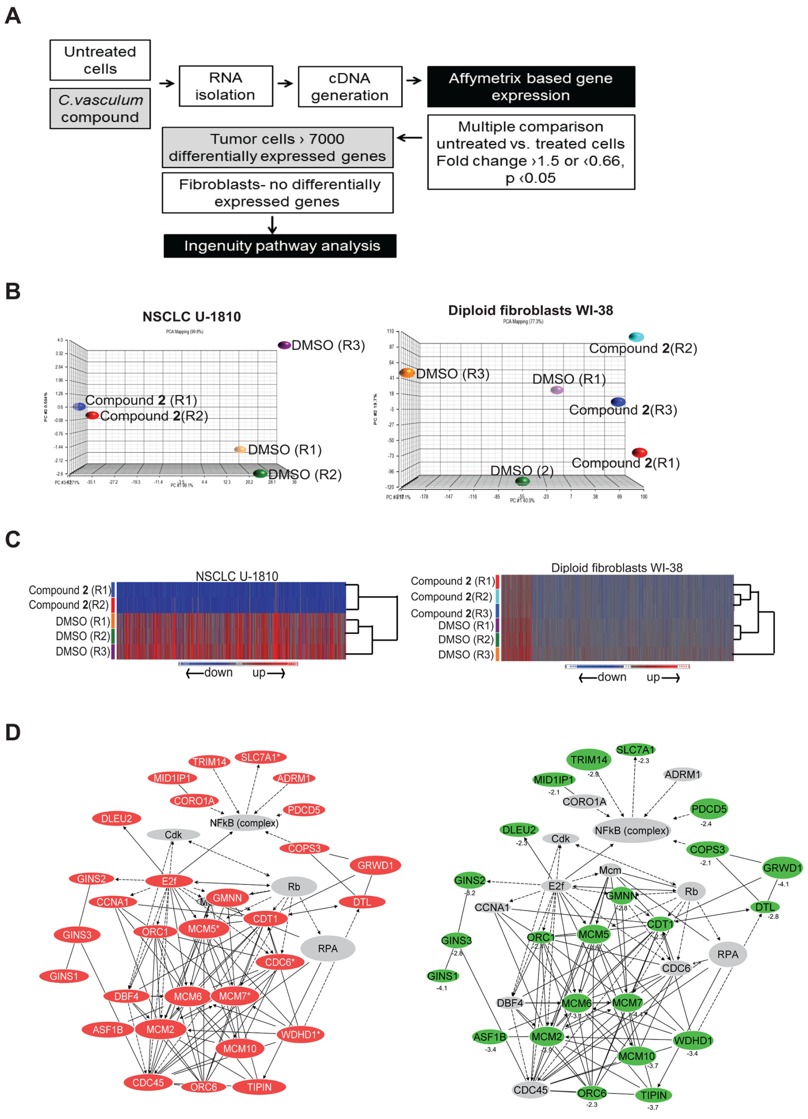

Supplementary Figure S1: Gene expression analysis of NSCLC U-1810 and fibroblasts WI-38, treated with compound 2 (3 µmol/L) for 24 hours reveals that change in gene expression is induced only in tumor cells. A. Overview of gene expression experiment: 2 replicates of NSCLC U-1810 cells and 3 replicates of WI-38 diploid fibroblasts were treated with 3 µmol/L concentration of compound **2**. **B.** PCA visualizing the variance in the NSCLC U-1810 or diploid fibroblasts WI-38 treated with compound **2** (blue, light blue and red) or DMSO (violet, orange and green). 3 principal components, visualizing 99.8% of the variance in the data is shown. **C.** Heat map and dendrogram of hierarchical clustering of NSCLC U-1810 or diploid fibroblasts WI-38 treated with compound 2 (blue, light blue and red) or DMSO (violet, orange and green). **D.** A gene signature of genes upregulated in breast cancer cells treated with IGF-1 was taken from Creighton et al [39]. The genes were loaded into IPA and different networks were generated. The top tanked network was selected and the gene expression data from NSCLC U-1810 treated with compound **2** (see (A)) was overlaid. *Red*: upregulated genes; *green*: down regulated genes; *grey*: genes showing no alteration. *Left panel*: Gene alterations associated with IGF-1 in BC cells obtained from [39]; *Right panel:* NSCLC U-1810 treated with compound **2** as outlined in (A). The fold down regulation of the indicated genes in response to compound **2** as compared to DMSO treated cells is indicated.

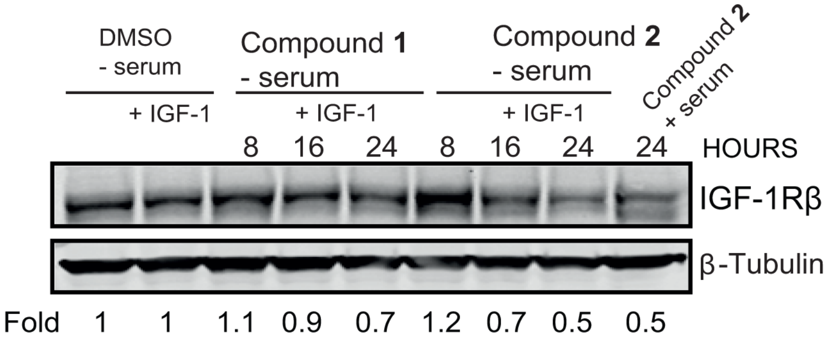

Supplementary Figure S2: IGF-1Rβ expression levels prior and post compound 1 and 2 exposure under serum starvation conditions. NSCLC U-1810 cells were deprived of serum overnight , exposed to IGF-1 (50 ng/ml) and treated with compounds as in Figure 1B and total IGF-1Rβ expression analyzed by Western blotting with β–Tubulin as marker of loading. Lane 9 contains NSCLC U-1810 cells treated under serum conditions to enable comparison. Resulting bands were scanned by densitometry and the fold to DMSO-treated cells is given after correction for loading differences.

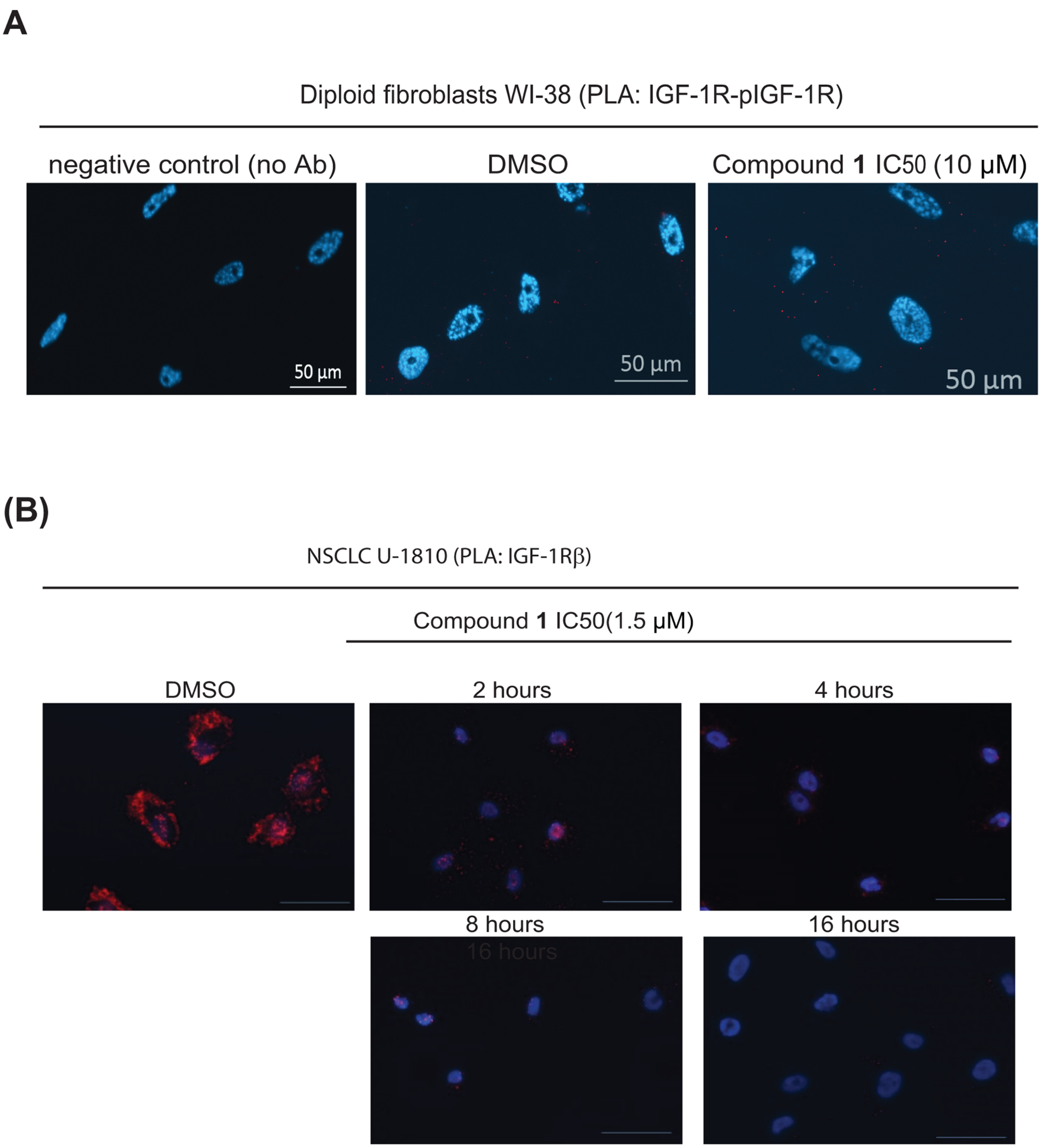

Supplementary Figure S3: Proximity ligation assay demonstrates no inhibition of phosphorylation of IGF-1R β in diploid fibroblasts but a dose dependent degradation of IGF- IGF-1R β in tumor cells upon treatment with compound 1. A. Diploid fibroblasts WI-38 were exposed to IC_{50} of compound **1** (10 μmol/L) for 24 hours and stained with phospho-IGF-1R β (Tyr1135/1136) and total IGF-1R interacting antibodies by PLA (red) and with DAPI staining for nuclei (blue). As a technical control for PLA, DMSO sample without the primary antibodies was used. Scale bars, 50μm. **B.** NSCLC U-1810 cells were exposed to compound **1** (IC_{50}) for 2, 4, 8 and 16 hours and stained with two IGF-1R β interacting antibodies in PLA (red) and with DAPI staining for nuclei (blue). Scale bars, 50μm.

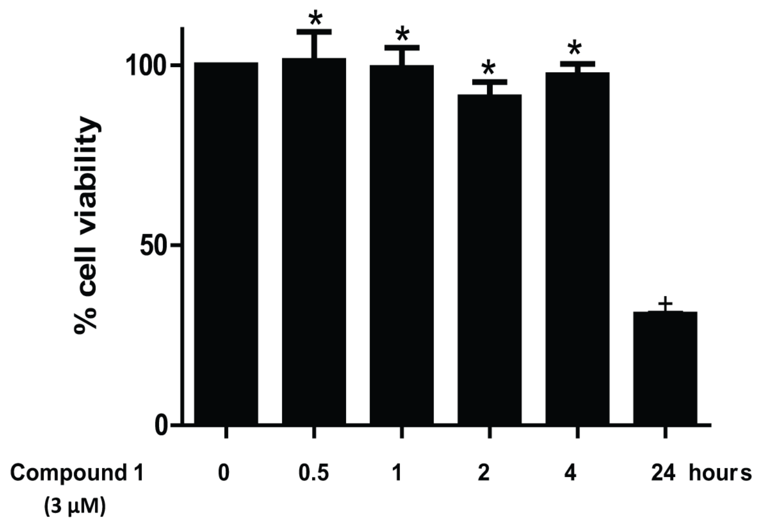

Supplementary Figure S4: Short term exposure of U-1810 cells to compound 1 show no toxicity. U-1810 cells were exposed to3 µmol/L of compound **1** for 0.5, 1, 2, 4 and 24 hours. At the end of continuous exposure, cell viability was examined using MTT cell viability assay. Cell viability is given as the percentage of cell survival compared to DMSO solvent–treated cells. The mean of three independent experiments ± SE is given. *p<0.01 comparison with 24 hours treated.

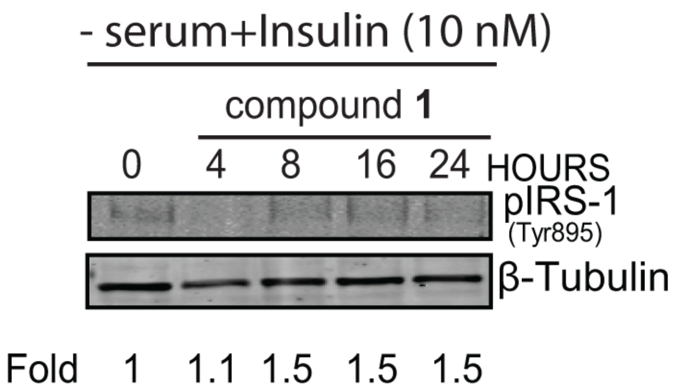

Supplementary Figure S5: phospho-IRS-1 levels prior and post compound 1 treatment in serum starved insulin treated NSCLC cells. NSCLC U-1810 cells were cultured in serum free media overnight and exposed to Insulin (10 nM) for 15 min after which compound **1** (IC_{50} 1.5 µmol/L) was added for 4, 8, 16 and 24 hours respectively. Phosphorylation of IRS-1 was analyzed and β–Tubulin was used as loading control. Resulting bands were scanned by densitometry and the fold to DMSO-treated cells is given after correction for loading differences.

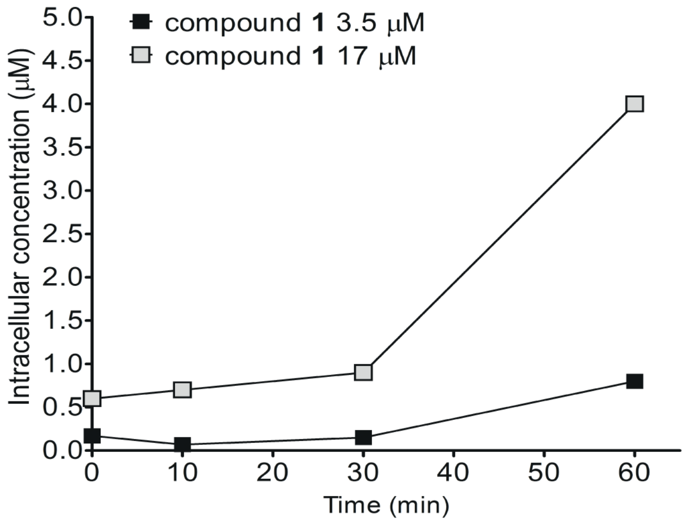

Supplementary Figure S6: Intracellular concentration of compound 1. NSCLC U-1810 cells were exposed to 3 and 17 μmol/L of compound **1** for indicated time. The concentration of compound **1** in the cells was determined by LC-MS (APCI). Graph represents concentration of compound **1** inside the cell at the indicated time points.
